# Cost analysis of youth clinic network in Estonia

**DOI:** 10.1186/s12978-015-0025-8

**Published:** 2015-05-01

**Authors:** Jari Kempers

**Affiliations:** Qalys Health Economics, Middenweg 239 I, 1098AP Amsterdam, the Netherlands

**Keywords:** Adolescent, Youth-friendly, Youth clinic, Sexual and reproductive health, Cost

## Abstract

**Background:**

Youth-friendly sexual and reproductive health (YFSRH) services for young people have high priority in many countries. Yet, little is known about the actual cost of delivering YFSRH services. This article analyses costs of a fully scaled up national youth clinic network (YCN) in Estonia. It reports; 1) total budget of the YCN during the period 2002–2012, and 2) annual clinic level costs of three youth clinics (YCs) in 2012.

**Methods:**

The retrospective cost analysis is based on financial and medical records of Estonian Health Insurance Fund (EHIF), Estonian Sexual Health Association (ESHA), National Institute for Health Development and the YCs. The programme level costs are analysed per year, financing source and a portion spent on coordination in 2002–2012. Costs of three YCs are analysed per clinic, expense category, patient and healthcare service in 2012.

**Results:**

The total budget of the YCN was €8.38 million and it served 304,000 young patients in 2002–2012. 95% of the total budget was financed by the EHIF. 3.6% was spent on coordination. The YCs in Tallinn, Tartu and Ida-Virumaa had annual budgets of €247,000, €267,000 and €42,000 respectively. In 2012 the three YCs provided YFSRH services to 19,700 patients, excluding sexuality education lessons and internet counselling. The YFSRH services cost €543,000. Consequently, the average cost per patient was €27.76. The largest expense categories were personnel salaries 35% and medical supplies 33%. Cost of the YFSRH services were; STI consultation €54.80, SRH counselling €13.13, contraception consultation €9.32, internet counselling €8.21 and sexuality education lesson €1.52.

**Conclusions:**

The Estonian YCN is a positive example for other countries considering or already implementing similar programmes. The cost analyses highlighted the following: Sustainable funding is particularly important, without it the YFSRH services would not have been scaled up and sustained on the national level in Estonia. Investment in professional coordination of the YFSRH services is recommended, and it does not necessarily have to be expensive. Only 3.6% of the total budget of YCN was used for ESHA coordination, which is a small portion especially when taking into account ESHA’s substantial contributions to development, training, quality improvements and representation of the YCN.

## Background

Youth-friendly sexual and reproductive health (YFSRH) services for young people have high priority in many countries. Yet, little is known about the actual cost of delivering YFSRH services. This article reports costs of a fully scaled up national youth clinic network (YCN) in Estonia. It calculates total budget of the YCN during the period 2002–2012, and costs per youth clinic (YC) and healthcare service in 2012.

The Estonian YCN is an important positive example for other countries considering implementation of similar YFSRH services programmes. The Estonian situation is interesting because: Firstly, sexual and reproductive health (SRH) outcomes of Estonian youth have improved remarkably since the early of 2000s. Secondly, the countrywide YFSRH services programme has been sustained with national funding over a decade. Policy makers and programme managers in other countries can use the Estonian example to support lobbying, planning and implementation of similar programmes.

The first YC of Estonia was started in 1991. In the following decade the Estonian YCN was successfully scaled up to the national level. Sexual health outcomes of Estonian adolescents and young people improved remarkably during the period 2001–2009. Annual abortions, sexually transmitted infections (STIs) and diagnosed HIV infections in the age groups 15–19 and 20–24 years old were reduced by 37%, 55% and 89%, respectively [1,2]. The YCN was implemented simultaneously with Estonian school-based sexuality education programme. Together these two interventions contributed to the improvement of SRH outcomes. This article is continuation of a policy analysis of success factors of scaling up of the Estonian YCN [3] and the World Health Organizations’ (WHO) case study on the network [4].

The objectives are to assess **programme level** costs during the period 2002–2012: 1) how much the national YCN programme cost? 2) how much was spent on coordination? 3) how the YCN was financed? Furthermore, to analyse annual **clinic level** costs in 2012: 4) what were the cost per YC? 5) what were the main expense categories? 6) what was the average cost per patient? And 7) what were the cost of SRH services?

### Youth clinic network in Estonia

Estonia is the northernmost of the three Baltic countries. In 1991 Estonia regained its independence from the Soviet Union. In 2004 the country became a member of European Union. Population of Estonia is 1.29 million inhabitants, of which 12% (155,000 persons) are aged 15–24 years [5]. 69% of the population are Estonians and Russians are the largest minority (24.8%) [5].

Development of the Estonian YCN had three distinct phases; 1) grass root initiatives in 1991–1994, 2) establishment of the YCN in 1995–2001, and 3) sustainable financing and professional management in 2002–2012. The cost analyses cover the last period. *Grass root initiatives in 1991–1994*. In the beginning of 1990s teenage pregnancy rates and STIs incidence were high among Estonian youth. A group of Estonian enthusiastic gynaecologists wanted to create a YCN in Estonia, similar to a youth clinic network in Sweden [4]. The first Estonian YC was founded in Viljandi in 1991, followed by clinics in Tallinn and Tartu. At the end of 1994 five YCs had been established. Estonian Sexual Health Association (ESHA) was also founded in 1994. *Establishment of the YCN in 1995–2001*. A network of YCs was established during this period. ESHA started coordinate the network, organize personnel trainings and publish sexuality education materials. At the end of 2001 there were 15 YCs. *Sustainable financing and professional management in 2002–2012*. During the first decade (1991–2001) the YCs struggled with their financing, but the situation changed when Estonian Health Insurance Fund (EHIF) started to finance the YCs and ESHA in 2002. In the beginning of 2000s the primary focus of the YFSRH services was to tackle raising HIV epidemic in Estonia. Other priorities were to improve the quality of services and increase management capacity. The sustainable financing from EHIF helped the YCN to focus on these improvements.

There were 18 YCs in 2012. The YCs are; departments of larger healthcare institutions, private gynaecological practices, or private healthcare companies. All the clinics offer free services to young people up to the age of 25 years in Estonian and Russian. Most of the YCs are open daily and serve exclusively young people. All the YCs must comply with the quality requirements and operational principles of the YCN, which define: 1) objectives of YCs, 2) operational principles, 3) provided SRH services, 4) target groups, 5) quality requirements, and 6) monitoring and evaluation indicators [6]. In 2012 YCs’ service package included the following SRH services:

1. STI consultations; STI testing, treatment and follow-up consultations, HIV services; voluntary confidential counselling and testing and in case of an HIV positive result referral for specialist consultation.
2. Contraceptive consultations; information and counselling about contraceptives, prescription and renewal of contraceptive prescriptions, and insertion of contraceptive devices.
3. SRH counselling; counselling, pregnancy diagnostics and referral for antenatal care or safe abortion, psychological counselling, human papilloma virus vaccination and counselling, and a range of other SRH services.
4. Sexuality education lessons at schools or YCs.
5. Website and internet counselling. ESHA manages a youth counselling website [www.amor.ee]. On the website young people find information about puberty, intimate relationships, sexuality, pregnancy, contraceptives and STIs. The website offers internet counselling services. Young people can ask anonymous questions about the above topics in Estonian or Russian. The questions are answered by professional personnel, such as youth counsellors, social workers, psychologists, sexologists or medical doctors.

The YCN, as its name describes, is a network of youth clinics. Estonian Sexual Health Association (ESHA) is a non-governmental organization, which coordinates and represents the YCN. ESHA also accepts of new YCs to the network. However, there is no management relationship between ESHA and the YCs. ESHA and YCN form an umbrella organization for the YCs. The YCs are a part of Estonian healthcare system, which is governed by the Ministry of Social Affairs.

ESHA played a crucial role in development and scale-up of the YCN. ESHA defines and oversees the quality of YFSRH services. The YCs report quarterly; visitor data, medical tests and procedures, diagnosed STIs and pregnancies, to ESHA. It provides training for personnel of the YCs. Moreover, ESHA represents and advocates the YCN to policy makers, EHIF and other stakeholders. Since 2002 EHIF is the main financier of the YCs. A main turning point was passing of the Health Insurance Act in the Parliament of Estonia in 2002 [7], which enabled EHIF to start finance disease prevention and health promotion programmes, like the YCs and ESHA. Each YCs and ESHA are separately contracted and financed by EHIF. The financing mechanism is based on the number of YFSRH services delivered by each clinic. As a result, both medical and financial records are well-documented. EHIF pays the YCs fixed fees for each of the YFSRH services (STI-, contraception consultations and SRH counselling). The fees are fixed regardless of the duration of consultation or counselling session. EHIF calculates the reimbursement prices for each YFSRH service type from average cost of working time, laboratory tests, medical and other materials used per one patient. In addition the YCs receive funding from the National Institute for Health Development (NIHD) for uninsured patients, and from local authorities and international organizations for SE lectures and training. More detailed description of the YCN can be found in the WHO studies [3,4].

### Methodology

The retrospective cost analysis is divided into two sections: 1) programme level costs of the YCN in 2002–2012, and 2) clinic level costs of the three YCs in 2012. The cost analysis was carried out from healthcare provider’s perspective. All the costs related to delivery of the YCN program were included in the analyses, regardless who financed the services. The primary source of cost and SRH service data were reimbursement records of EHIF. Documentation of NIHD, EHSA and YCs were also used. Support received from local and international organizations were also included. All costs are presented at 2012 prices. Historical cost data were adjusted for inflation [8]. Amounts in Estonian kroons were translated to Euros at a rate of 15.65 [9]. The results were compared with a costing study on similar YFSRH services in Moldova [10]. To enable comparison, Euro amounts were first converted to USD [11] and then costs of both programmes were adjusted for purchasing power parity of each country [12].

### Programme level costs in 2002–2012

Financial records of the initial start-up period in 1991–2001 are poorly documented. Consequently, cost analysis of the early years of YCN was not feasible. The situation changed in 2002, when EHIF started to finance the YCs and ESHA. Since then good centralized data on reimbursement payments and SRH services are available. The time horizon of the programme level cost analysis is 2002–2012. The analysis includes all SRH services and SE lessons provided by all YCs, and centralized internet counselling services during this period. Total budget of the YCN, and ESHA’s coordination costs were calculated for the period 2002–2012. A breakdown of financing sources of the YCN is provided for the same period.

### Clinic level costs in 2012

Three YCs out of 18 YCs in Estonia were selected for the clinic level cost analysis. The selection was based on the following criteria; 1) the primary focus of the YC is youth counselling and SRH services, 2) it provides service package described in the quality requirements guideline [6], 3) it scored higher than 3.63 on a client satisfaction survey in 2007 [13], and 4) the YC is contracted by EHIF. The two largest YCs, Tallinn Sexual Health Clinic and Tartu Sexual Health Clinic, fulfil the criteria. According to EHIF’s reimbursement records these two YCs cover 57% of all patients of the YCN. Therefore the two largest YCs were an obvious choice for the study. Furthermore, a smaller Ida-Virumaa YC in Jõhvi was selected. Table 1 summarizes medical personnel, opening hours, locations, catchment populations and patients of the three YCs in 2012.

**Table 1:** **Characteristics of the selected three youth clinics in 2012**

**Youth clinic**	**Medical personnel, full-time equivalents (FTE)**	**Opening hours**	**Location**	**Catchment population 15–24 years**	**Patients**
Tallinn Sexual Health Clinic (*Tallinn Seksuaaltervise Kliinik)*	Gynaecologists 1.60, Medical doctors 0.20, Midwifes 2.75, Psychologist 0.13, Total FTEs 4.68	45 h per week, Mon - Fri 8–17	Tallinn - the capital and the largest city, with three youth clinics.	16,000	8,700
				one-third of the age group in Tallinn	
Tartu Sexual Health Clinic (*Tartu Seksuaaltervise Kliinik)*	Gynaecologists 1.00, Medical doctors 1.60, Midwifes 0.70, Nurses 1.40, Psychologist 0.30, Total FTEs 5.00	40 h per week, Mon 9–19, Tue - Thu 9–17, Fri 9–15	Tartu - second largest city, with a large university.	16,000	9,400
Ida-Virumaa Youth Counselling Centre (*Ida-Virumaa Noortenõustamis-keskus)*	Gynaecologists 0.40, Medical doctors 0.04, Midwifes 0.20, Total FTEs 0.64	21.5 h per week, Mon 9–16.30, Tue - Fri 13–16.30	Jõhvi - a smaller town with many villages in the catchment area.	3,000	1,300

The clinic level costing was conducted for year 2012, which was the closest complete financial year at the time of conducting this study. First, *cost per YC* was calculated. Second, total costs were divided into six *expense categories*: 1) salaries, 2) medical supplies, 3) operations, 4) personnel training, 5) ESHA coordination, and 6) internet. The salary costs relate to gross salaries of personnel of the YCs. The medical supplies include costs of laboratory tests (such as smears, STI tests and pregnancy tests), medicines for emergency care and medical materials used in the YCs. Patients’ medicine costs at pharmacies were excluded. The operation costs include rent, facilities and maintenance of the YCs. This also covers computers, office supplies and transportation costs. The personnel training costs cover training of employees of the three YCs. The ESHA coordination cost relate to YCs’ portion of gross salaries of ESHA personnel and procurement of information materials. The number of patients reached per YC was used as a proxy for allocation of the ESHA coordination costs. The internet costs relate to website and internet counselling services. YCs’ portion of these costs was allocated on the basis of number of inquiries from the catchment area of each clinic. The inquiries from Tallinn area were divided by three, because the city has three YCs. Third, *cost per patient* was calculated. The cost per patient calculations include all SRH services and exclude SE lessons and internet counselling. Allocation of the overhead costs was based on the number of SRH services provided by each YCs in 2012. EHIF’s reporting system does not capture if the same patient visited an YC several times in a year. Consequently, the cost of reaching a patient must be interpreted as an approximation. Fourth, *cost of SRH services* were calculated. In EHIF’s reporting system, SRH services are grouped and coded in the following three categories; 1) *STI consultations*, 2) *contraception consultations*, and 3) *SRH counselling*. Personnel of the YCs can record a consultation or a counselling session to only one of these international classification of diseases (ICD) codes. They choose which code is the most appropriate. Consequently, the data does not capture if a patient had several treatments or tests during the same visits, for example an STI- and a pregnancy test. Two additional service categories were used; 4) *Sexuality education lessons* given by personnel of the YCs. Duration of one lesson is 2 × 45 min. Additional 30 min was reserved for preparation of a lesson. Average class size was 15 students [14]. And 5) *Internet counselling,* which includes answers to inquiries from the catchment areas of the three YCs in 2012.

## Results

### Programme level costs in 2002–2012

#### How much the national YCN programme cost in 2002–2012?

**Figure 1:**
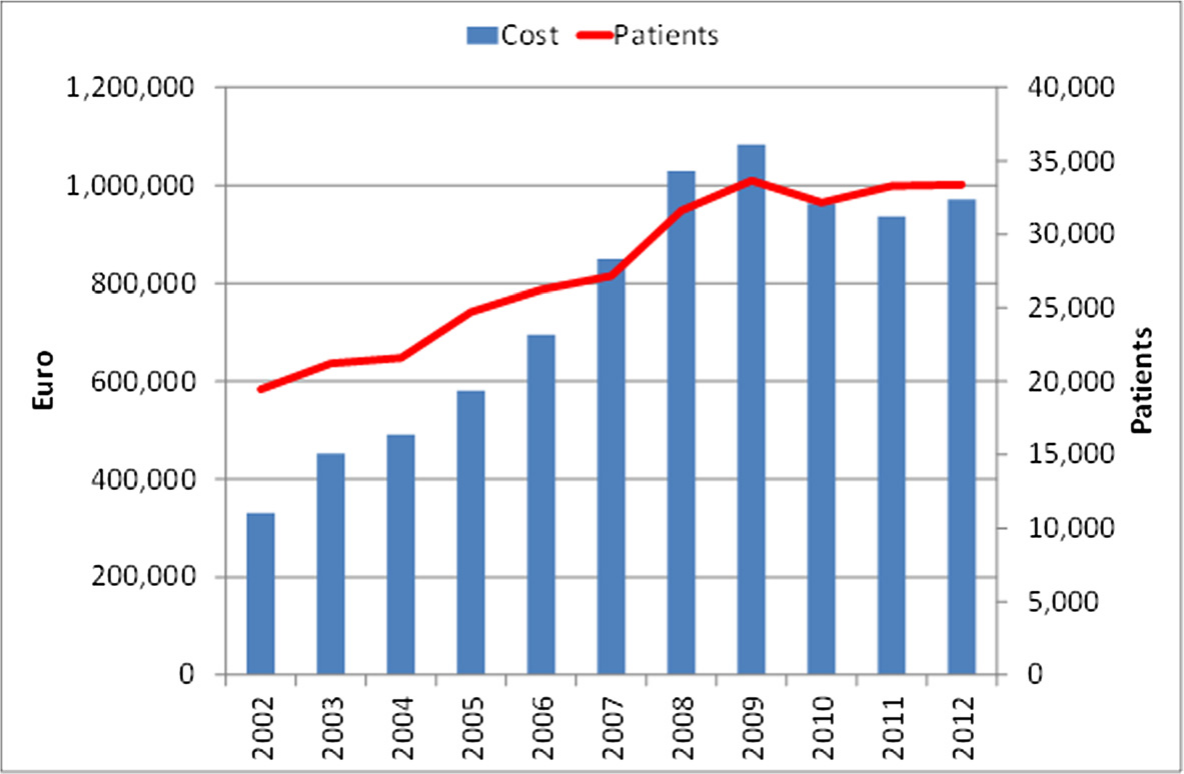
Annual budget of the youth clinic network in 2002–2012.

Figure 1 shows the annual budgets (bars) and patient numbers (line) of the YCN during the period 2002–2012. The funding of YCN increased gradually from €330,000 (15 YCs) in 2002 to the maximum of €1,080,000 (19 YCs) in 2009. During the period 2002–2009 YCs’ expenditure grew faster than the number of patients, because reimbursement prices of some SRH services were increased and new services were added to the health insurance package. In 2009 the economic crisis forced EHIF to lower some reimbursement prices. Since then annual budget of the 18 YCs levelled to approximately €950,000. The cumulative total budget of YCN was €8.38 million in the period 2002–2012. During the same period the YCN served 304,000 patients (excluding SE lessons and internet counselling).

#### How much was spent on coordination of the YCN in 2002–2012?

€299,000 were spent on coordination carried out by ESHA during the period 2002–2012. This represents 3.6% of the total budget of the YCN during the same period.

#### How the YCN was financed in 2002–2012?

**Figure 2:**
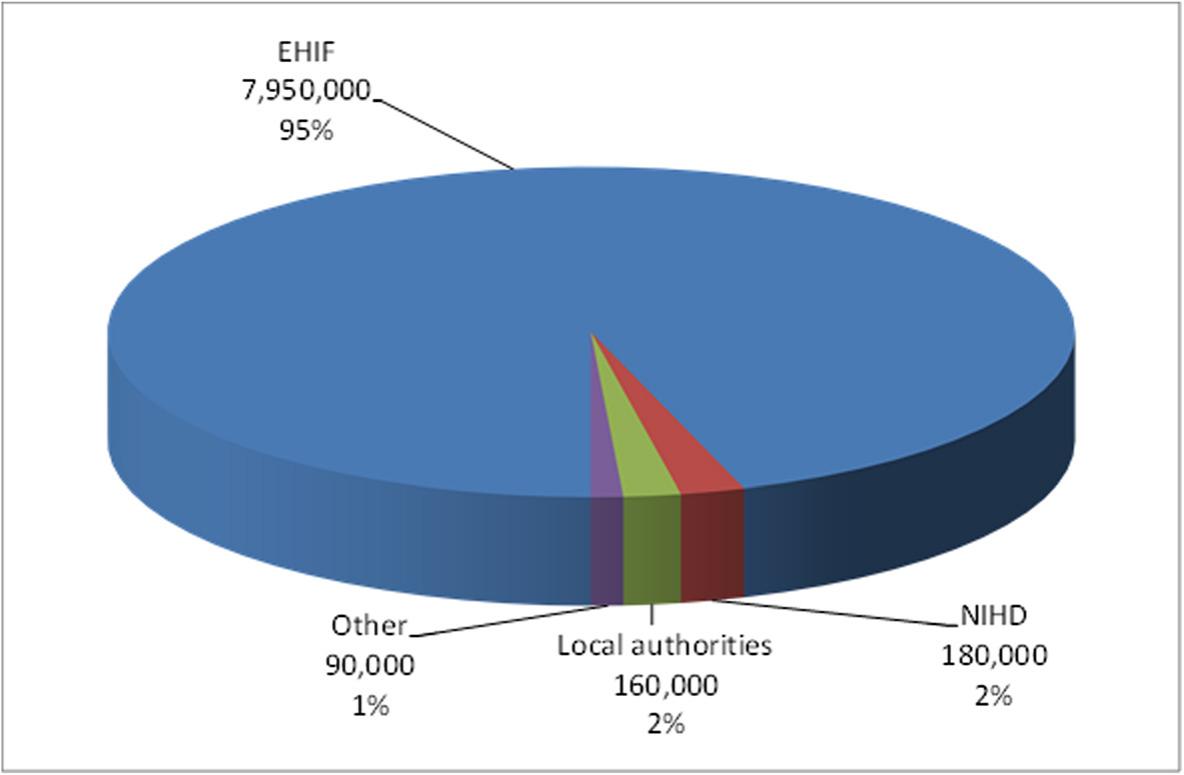
Financing sources of the youth clinic network in 2002–2012.

Figure 2 provides a breakdown of the total budget of YCN (€8.38 million) per financing source in 2002–2012. EHIF was by far the largest financier. It financed 95% (€7.95 million) of the total budget. NIHD payments for uninsured patients accounted for 2% (€180,000). Contributions from local authorities were 2% (€160,000) and support from other sources 1% (€90,000).

### Clinic level costs in 2012

#### What were the cost per YC?

In 2012 annual budget of all 18 YCs were €970,000. The two largest YCs, Tallinn and Tartu, had annual budgets of €247,000 and €267,000 respectively. The smaller Ida-Virumaa YC had a budget of €42,000. Together the three YCs accounted for €556,000, which is 57% of the annual budget of the YCN in 2012.

#### What were the main expense categories?

**Figure 3:**
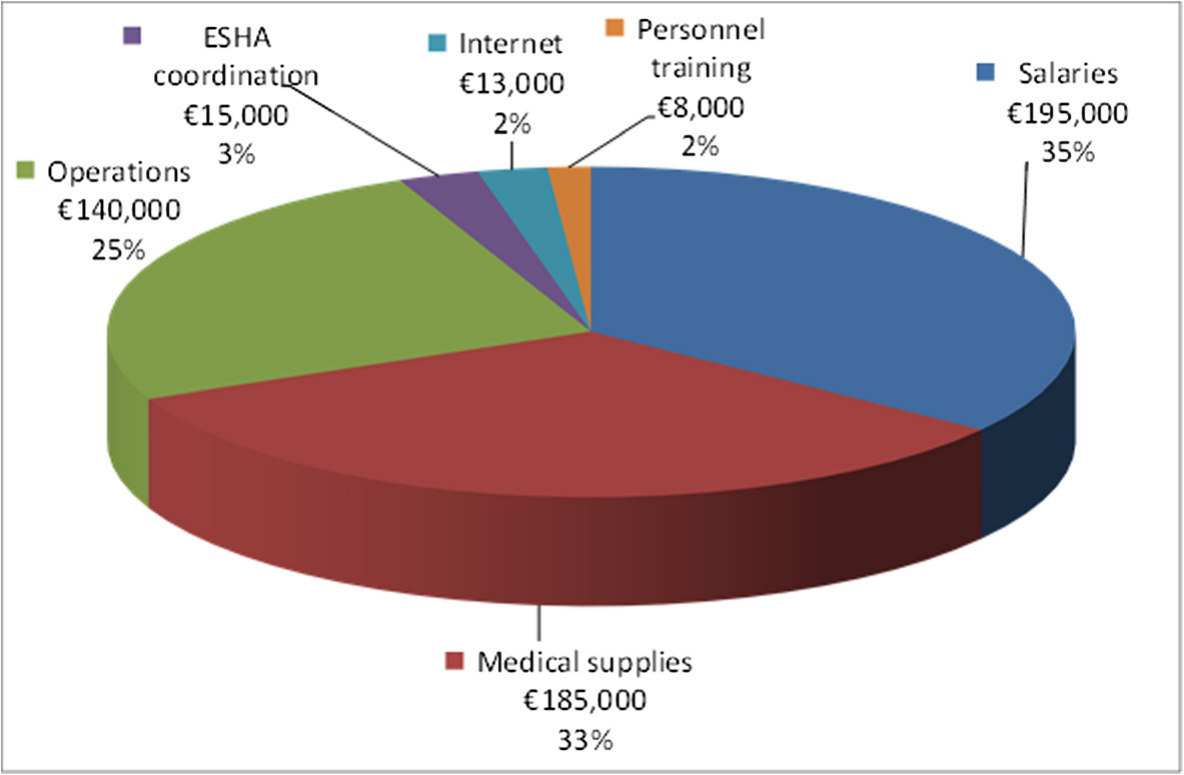
Expense categories of the three youth clinics in 2012.

Figure 3 provides an overview of expenses of the three YCs in 2012. Personnel salaries were the largest expense category 35% (€195,000). The second largest group was medical supplies 33% (€185,000). Third were operation expenses 25% (€140,000). 4% (€15,000) was allocated to coordination costs of ESHA. Finally, three YCs’ portion of costs of centralized internet counselling services and personnel trainings were 2% (€13,000) and 2% (€8,000) respectively.

#### What was the average cost per patient?

The total cost of SRH services provided by the three YCs in 2012 were €543,000. This amount includes direct EHIF reimbursement payments for the SRH services and allocated overhead costs, and excludes costs of SE lessons and internet counselling. The three YCs provided SRH services to 19,700 patients. Consequently, the average cost per patient was €27.76 in 2012.

#### What were the cost of SRH services?

Costs of SRH services delivered by the three YCs in 2012 are summarized in Table 2. The total costs column shows the total cost of each healthcare service category. The services column summarizes the number of services provided by the three YCs in 2012 and the last column the unit cost per SRH service. STI consultations were the most expensive services, at an average cost of €54.80. The cost are high because STI management often requires a combination of services, for example consultation, laboratory test and follow-up consultation. SRH counselling cost on average €13.13. Contraception consultations had an average cost of €9.32 per consultation, which are lower because 58% of these consultations are shorter appointments for renewals of contraceptive prescriptions. Internet counselling cost on average €8.21 per responded inquiry. This is relatively expensive, because answering individual questions in writing is labour-intensive. A SE lesson taught by personnel of the YCs was €1.52 per student, which is low because the SE lessons are given in groups.

**Table 2:** **Total costs, number of SRH services and unit cost per SRH service in the three youth clinics in 2012**

**Healthcare service**	**Total costs**	**Services**	**Unit cost per service**
STI consultation	^*^€411,000	7,500	€54.80
SRH counselling	^*^€63,000	4,800	€13.13
Contraception consultation	^*^€69,000	7,400	€9.32
Internet counselling	^†^€11,000	1,340	€8.21
Sexuality education lessons	^††^€2,000	1,320	^†††^€1.52

*Direct EHIF reimbursement payments and allocated overhead costs. ^†^Three YCs’ portion of cost of centralized internet counselling services. ^††^Cost of sexuality education lessons provided by the three YCs. ^†††^Cost per person reached, not cost per sexuality education lesson.

## Discussion

During the period 2002–2012 the youth clinic network served 304,000 young patients and its total budget was €8.38 million. 95% of this was financed by the EHIF. Only 3.6% of the total budget was spent on coordination carried out by ESHA. In 2012 the three YCs, in Tallinn, Tartu and Ida-Virumaa, had annual budgets of €247,000, €267,000 and €42,000 respectively. In 2012 the three YCs provided YFSRH services to 19,700 patients (SE lessons and internet counselling excluded). The YFSRH services cost €543,000. Consequently, the average cost per patient was €27.76. The largest expense categories were personnel salaries 35% and medical supplies 33%. Cost of the YFSRH services were; STI consultation €54.80, SRH counselling €13.13, contraception consultation €9.32, internet counselling €8.21 and sexuality education lesson €1.52.

Estonian Health Insurance Fund is by far the largest financier of the YCN. EHIF financed 95% of the total budget. The sustainable national funding has been crucial for the success of the YCN. Without the funding many of the YCs might not been able to continue or scale up their YFSRH services. This is an important example for other countries planning to implement similar YFSRH programmes. It is important to guarantee sustainable funding for YFSRH services. A stable funding environment allows programme managers to switch their focus from short-term financial survival to long-term planning, quality improvements and sustainable scale-up of the YFSRH services.

Benefits of the investment in professional coordination of the YCN are clear in Estonia. Only 3.6% of the total budget of YCN was spent on coordination carried out by ESHA. This is a small portion especially when taking into account substantial contributions of the association. ESHA played a key role in development, implementation and scale-up of the YFSRH services in Estonia. ESHA defines and oversees the quality of YFSRH services, and provides continuous training for YCs’ personnel as well. Moreover, ESHA played an important active role in advocating and promoting the YFSRH services to policy makers and medical professionals. ESHA’s repeated lobbying and fundraising efforts helped policymakers and EHIF directors to form a favourable opinion of the YCs. The Estonian example shows, to other countries planning or implementing similar programmes, that it is beneficial to have a specialized professional organization coordinating, overseeing service quality and representing YCs.

The results were compared with a costing study on similar YFSRH services in Moldova [10]. Euro amounts were converted to USD and adjusted for different price levels in the two countries. Average annual budgets of the three Estonian YCs were similar to budgets of four well performing youth clinics in Moldova. The Estonian YCs had 1.1 times higher budgets than the YCs in Moldova. SRH counselling and contraception consultations were also slightly more expensive in Estonia, 1.2 and 1.7 times more expensive respectively. SE lessons were 0.5 times cheaper in Estonia. The only larger difference were STI consultations, which were 7.7 times higher in Estonia. This is caused by the use of more expensive STI laboratory tests in Estonia.

### Limitations

The cost analyses have some limitations. Firstly, the early years (1991–2001) of the YCs and YCN are not included. Therefore the results do not represent cumulative total cost of the YCN, but the period of EHIF financing (2002–2012). Secondly, the clinic level cost analyses were done in three YCs. Consequently, the clinic level results might not represent the entire YCN. Thirdly, costs of YFSRH services are largely determined by reimbursement prices of the EHIF, as it is the main financier of the YCs. EHIF has great negotiation and decision power on the service prices. Hence, the costs of YFSRH services might not reflect the actual cost of delivering the services. Fourthly, the provided YFSRH services can be recorded in only one ICD code. Consequently, the data does not capture if a patient had several treatments or tests during the same visits, for example an STI- and a pregnancy test. Moreover, the data does not show if a patient had several follow up consultations or counselling sessions for the same health condition.

## Conclusion

The Estonian YCN is a positive example for other countries considering or already implementing similar programmes. The cost analyses highlighted the following: *Sustainable funding* is particularly important. In Estonia a main turning point was a legislation change in 2002 [7], which enabled EHIF to start finance disease prevention and health promotion programmes, like the YCs and ESHA. Since then 95% of the total budget of the YCN has been financed by EHIF. Importantly, a stable funding environment allows programme managers to switch their focus from short-term financial survival to long-term planning, quality improvements and sustainable scale-up of the YFSRH services. *Investment in professional coordination* of YFSRH services is recommended, and it does not necessarily have to be expensive. Only 3.6% of the total budget of YCN was used for ESHA coordination, which is a small portion especially when taking into account ESHA’s substantial contributions on development, personnel training, service quality improvements and representation of the YCN.
